# Machine Learning in Single-Molecule Tracking Analysis of Superresolution Optical Microscopy Data

**DOI:** 10.3390/cells15080686

**Published:** 2026-04-13

**Authors:** Lucas A. Saavedra, Francisco J. Barrantes

**Affiliations:** Division of Molecular Neurobiology, Biomedical Research Institute UCA-CONICET, Buenos Aires C1107AAZ, Argentina; lucasarielsaavedra@uca.edu.ar

**Keywords:** artificial intelligence, machine learning, deep learning, feature engineering, stochastic processes, single-molecule tracking, diffusion

## Abstract

**Highlights:**

**What are the main findings?**

- Motion of molecules in live cells can be studied via single-molecule tracking (SMT).
- SMT beyond the diffraction limit requires optical superresolution imaging.

**What are the implications of the main findings?**

- Machine learning facilitates and improves the analysis of SMTs.
- Molecular motion provides functionally important information on the dynamics of molecular constituents of the cell.

**Abstract:**

Machine learning (ML) is transforming the analysis of biomolecular data, holding significant promise for improving the efficiency and accuracy of microscopy image analysis and for studying the dynamics of molecules in live cells. As data-driven approaches continue to evolve, they may eventually replace traditional statistical methods that rely on conventional analytical methods. This review examines and critically analyses the state of the art of ML techniques as applied to various levels of data supervision in the analysis of dynamic single-molecule datasets obtained using superresolution optical microscopy. Collectively encompassed under the umbrella of “nanoscopy”, these methods currently comprise targeted techniques such as stimulated emission depletion (STED) microscopy and stochastic techniques like single-molecule localization microscopies (SMLMs), comprising photoactivated localization microscopy (PALM), DNA points accumulation for imaging in nanoscale topography (DNA-PAINT) microscopy, and minimal fluorescence photon flux (MINFLUX) microscopy. These techniques all enable the imaging of subcellular components and molecules beyond the diffraction limit, and some are additionally capable of studying their dynamics in real time, as reviewed here, using several ML techniques that facilitate motion analysis in two or three dimensions with qualitative and quantitative characterisation in the live cell. It is expected that the growing use of learning-based approaches in biological microscopy data processing will dramatically increase throughput and accelerate progress in this rapidly developing field.

## 1. Introduction

**Figure 1:**
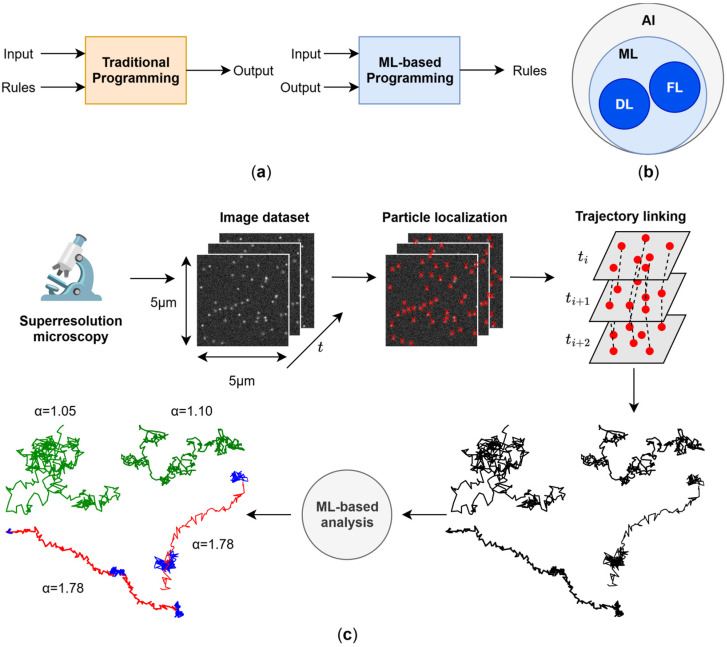
Analysis of superresolution microscope images in the era of ML. (**a**) Traditional programming involves the development of algorithms that contain rules about how to process inputs to return a specific output. However, defining such rules for complex tasks like classifying single-molecule trajectories and fitting them into the frame of theoretical models is difficult. ML changes the paradigm of traditional programming: instead of defining rules for a specific task, ML automatically finds the rules using recorded data. (**b**) AI includes ML, which can be further divided into deep learning (DL) and feature-based learning (FL). (**c**) Most superresolution microscopy experiments involve the extraction of single molecules from a raw image dataset to obtain a curated (validated) SMLM dataset which is further analysed to extract and characterise trajectories. Trajectory linking consists of linking localizations along successive frames at timestamps t_i_, t_i+1_, and t_i+2_ to obtain tracks. The resulting dataset is then used to obtain and analyse trajectories by linking localizations across adjacent frames. Both particle localization and trajectory linking can be accomplished via data-driven approaches. With ML-based analysis, extracted trajectories can be segmented, for example, into Brownian (green), superdiffusive (red), or confined (blue) regimes. Additionally, the anomalous diffusion exponent (α) can be calculated for each trajectory, characterising its average (anomalous) behaviour.

The concept of Artificial Intelligence (AI) can be traced back to 1950, when Alan Turing proposed the imitation game (later known as the “Turing test”) to test whether machines can think [1]. Although the term AI did not appear in Turing’s paper, it undoubtedly triggered the development of algorithms and models to tackle problems from a human perspective and, beyond this, attempt to surpass human performance. The definition of AI is still a highly debated issue, much like the challenge of defining intelligence itself [2]. A well-defined subset of AI is ML, the area of AI that combines mathematics, statistics, and computing to teach the latter to solve problems using ground-truth data without explicitly informing the computer how to perform the task through algorithms [3]. Traditional programming is based on developing detailed algorithms with manually defined rules about how to process data to return a specific output. However, such rules are often difficult to define for complex tasks (e.g., image classification). In ML, the rules are automatically defined using pre-defined outputs (Figure 1a). ML therefore differs from traditional programming, where explicit steps are crafted by the programmer to provide solutions [4]. ML can in turn be divided into two subsets (Figure 1b): feature-based learning (FL; predictive models that use manually derived features from data as input) and DL (representation learning-based prediction models that use raw data as input and automatically extract features) [5,6]. In the context of this review, a feature is a (discrete or continuous) characteristic of a specific object of the real world (e.g., the duration of a single-particle trajectory).

The impact of ML in biology is clearly reflected in the recent 2024 Nobel award in chemistry for the development of AlphaFold2 to predict the 3D structure of protein molecules using only the amino acid sequence [7]. This approach was later extended (AlphaFold3) to predict not only refined protein structures but also the complexes of these with ligands or other small molecules [8]. Many proteins—in particular membrane-bound proteins—are difficult to crystallise and the process is often time-consuming; some proteins cannot form 3D crystals at all. ML dramatically reduces the structure prediction to minutes, thus speeding up access to valuable structural information on biomacromolecules whilst attaining a resolution similar to that of experimental methods. In terms of the specific biological application of AI examined in this review, namely the analysis of single-molecule data obtained with superresolution microscopy of membrane proteins, the improvement in terms of speed and accuracy brought about by AI methods is particularly advantageous.

Optical microscopy has experienced a major breakthrough in the last two-and-a-half decades with the advent of superresolution (“nanoscopy”) techniques, enabling the imaging of cell structures beyond the diffraction barrier. Following the pioneer studies of Stefan Hell, William Moerner, Harald Hess, Eric Betzig and Mats Gustafsson, whose work gave rise to the field of superresolution microscopy, the armamentarium of techniques now covers a complex palette of conceptualisations, physical principles, and instrumental realisations. Nanoscopy methods encompass targeted approaches such as stimulated emission depletion (STED) microscopy (see review in [9]) or structured illumination microscopy (SIM [10,11]) on the one hand, and stochastic techniques covered under the term single-molecule localization microscopy (SMLM) (reviewed in refs. [12,13,14]) on the other. SMLM includes photoactivated localization microscopy (PALM) [15], transient binding of short fluorescently labelled oligonucleotides (DNA-PAINT, a variation of point accumulation for imaging in nanoscale topography) microscopy [16,17], reversible saturable optical fluorescence transition (RESOLFT) nanoscopy with photoswitchable fluorescent proteins [18,19] and minimal fluorescence photon fluxes (MINFLUX) microscopy [20].

In addition to the imaging process proper, nanoscopy requires extensive offline computer-driven intervention in image pre-processing steps, usually to remove artefacts or enhance the quality of the superresolution images for in-depth analysis, and a growing number of sophisticated analytical tools for post-processing the raw datasets and additional analysis of physical models to interpret the data (Figure 1c). The traditional analysis of static and dynamic single-molecule data is in many cases limited to averages of molecules at the population level because single-molecule analyses are often affected by biases and scarcity of data [21]. The qualitative and quantitative analysis of biomolecular data plays a key role in elucidating the distribution and function of proteins or other biomolecules that inhabit the cell. Addressing the dynamics of biomolecules in live cells adds another layer of complexity because of the spatio-temporal accessible windows [22,23] and the inherent photobleaching and phototoxicity side-effects [24,25] of nanoscopy methods. Fortunately, the development of AI-based approaches to undertake in-depth post-processing analyses of microscopy data is on the rise, introducing new analytical paradigms to unravel the structure and dynamics of molecular constituents of the cell.

The reader is referred to additional overviews on the foundations and implementation of nanoscopy techniques like STED [25], STORM or PALM [12,13,26,27], RESOLFT [18,28] or DNA-PAINT [17,25,29]; ancillary techniques like expansion microscopy [30,31,32]; superresolution methods for the cell biologist [33,34,35,36] and image processing and analysis [37,38,39,40]; and combinations thereof (e.g., DNA-PAINT with Click chemistry [41]; STORM and DNA-PAINT [42]; or DNA-PAINT combined with MINFLUX [43,44,45]).

Historically, the initial applications of optical superresolution methods in the field of biology were restricted to imaging fixed specimens beyond the diffraction limit, with the aim of obtaining detailed high-resolution structural information on cells. Imaging dynamic cellular phenomena obviously developed more slowly because of the above-mentioned challenges associated with the temporal resolution of camera-based acquisition, photobleaching due to laser illumination, and the limited time window that some buffers (e.g., those used in STORM imaging) could afford on live cells. Today, the boundaries between these two apparently contrasting foci have blurred. Current approaches to single-molecule localization and dynamic tracking require, and achieve, high-precision (sub-nanometre) localization for tracking individual molecules with high time (microsecond) and spatial (below the nm) windows [46], thus making this conceptual distinction obsolete.

The focus of this review is the application of ML in single-molecule tracking (SMT), outlining its advantages and limitations. ML-based techniques related to the pre-processing of superresolution images are not dealt with in this work. In Section 2 we analyse DL models for extracting localisations and trajectories from superresolution images; Section 3 reviews ML techniques for analysing the dynamics of single molecules; and in Section 4 we discuss prospects for the development of new ML-based tools in the superresolution SMT field.

## 2. Single-Molecule Localisation and Trajectory Linking

### 2.1. Single-Molecule Localisation in Dynamic Samples

Once microscopy images are experimentally obtained, stored, and pre-processed with traditional or ML methods, single-molecule positions must be extracted prior to further analysis. This process results in SMLM datasets containing a list of coordinates (and eventually additional information like intensity) about detected and validated molecules. Traditionally, localising single molecules in optical superresolution images relies on fitting a point-spread function (PSF, modelled as a Gaussian function) to the fluorescence-emitting source in multiple sub-regions of interest (ROIs) containing a low and sparse number of molecules [47,48]. However, these and similar processes are algorithmic, and they work when their assumptions are correct; furthermore, they are prone to user biases [49]. In addition, a high density of emitters makes particle localisation difficult because of the overlap of PSFs, an issue where traditional methods do not perform accurately. Using synthetic data mimicking real experimental conditions, ML-based methods bypass these and other biases and accelerate inference times. Moreover, most ML-based methods are parameter-free, thus dispensing with the need for time-consuming and subjective parameter selection procedures. Table 1 compares traditional and ML methods for particle localisation and tracking.

**Figure 2:**
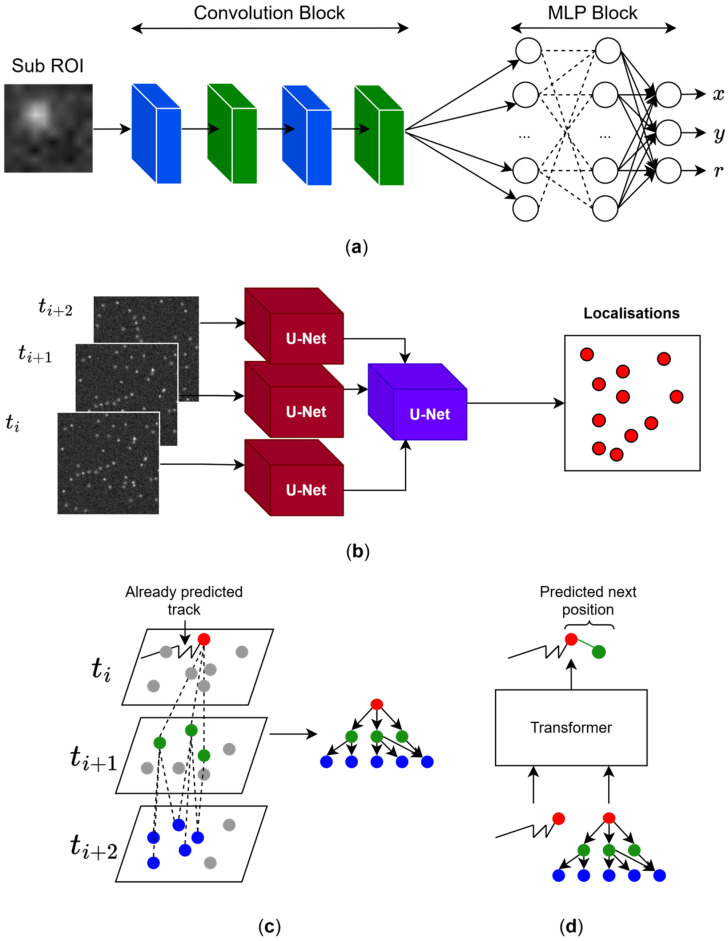
Particle localisation and tracking using ML-driven SMT analysis. (**a**) A CNN network is employed to predict the x,y coordinates and the distance of the emitter from the centre (r) in sub-ROIs [49]. The sub-ROI is used as input of the network, which is processed first by a convolution block. The blue and green layers correspond to standard convolutional layers and max pooling layers, respectively. Next, the features extracted by the convolution block are processed by an MLP block which makes the final prediction. The dashed lines represent the connections of the neurons of the first layer to those of the next layer, and the ellipse indicate that a layer can be composed of an undefined number of neurons. (**b**) Instead of relying on a single frame, a multi-layer network (like DECODE, SRST, or LiteLoc) can take several consecutive frames to infer, among other variables, the position of the particles. In the case of DECODE, multiple U-Net networks are set in parallel to analyse each consecutive frame. (**c**) MoTT builds spatio-temporal trees for each localisation close to the already predicted track. (**d**) Next, the tree is fed into a Transformer network with the current track to predict the next position. All microscopy frames in the figure were generated using ThunderSTORM [48].

Most ML methods for localising particles in biological material are intended for fixed samples. Giovanni Volpe and coworkers proposed to localise molecules in single frames using Convolutional Neural Networks (CNNs) [5,6], where a single frame is used as input (only one molecule is observed), and the network returns the molecular coordinates [49] (Figure 2a). Because the network processes single frames, it can be used to localise particles in dynamic samples. CNNs learn to extract useful features from frames. The root-mean-square error (RSME) for matching points in each frame decreases as the Signal–Noise Ratio (SNR) of the frame increases. With noisy frames (SNR = 1), RMSE is about 1 pixel, maintaining the same value across different emitter densities (number of fluorophores in the same ROI), thus reflecting its robustness under different experimental conditions. Although this particle localiser can be adapted to identify more than one molecule at a time [49,57], it does not consider particle blinking as in SMLM. Because particle blinking is an event that occurs in a few consecutive frames, neural networks should accept such frames as a single localisation.

Other works for single-molecule localisation found in the literature are primarily suited for static, structural analysis of SMLM data because they use synthetic data of immobile particles and perform inference over multiple frames rather than a single frame. However, these methods can be easily adapted to dynamic samples. First, the simulation procedures used to train and validate the methods can be modified to include molecular motion (e.g., free diffusion). Second, the use of multiple frames can be leveraged to localise the particles’ positions in the central frame of each sequence of frames. The only disadvantage of these modifications is that it is not possible to extract localisations from the first and last frames of an entire movie.

Ries and coworkers pioneered the use of complete and consecutive frames as input with DECODE, a multi-layer DL architecture based on concatenated U-Nets (a set of concatenated CNNs [58]) and trained on simulated data to extract not only particle positions, but also their uncertainties and frame background on 2D and 3D data using consecutive frames of stochastic SMLM superresolution data [59] (Figure 2b). At variance with Deep-STORM [60,61] and traditional localisation algorithms, DECODE uses simulations to train networks, requiring only simulated single frames containing intermittently blinking molecules at different time intervals, thus dispensing with the need to adapt algorithms to different experimental scenarios. DECODE also provides adaptable Jupyter Notebooks for training, enabling non-expert users without programming experience to use the software. Localisation error and detection accuracy improve when more than one frame is fed into DECODE, whereas accuracy decreases as the emitter density increases, indicating less robustness in this respect compared to sub-ROI analysis [49]. DECODE attains a detection accuracy (Jaccard index, JI) and localisation error of ~80% and 20 nm, respectively, with densities below 1 emitter per µm^2^. Above such limit, JI and localization error were <60% and >20 nm, respectively. On the downside, DECODE does not consider spatio-temporal relationships between consecutive frames, which are essential for dynamic experiments.

A recent improvement over DECODE is Super-Resolution SpatioTemporal (SRST) information integration, a multi-layer network that integrates Bidirectional ConvLSTMs to extract sequential information in both temporal and spatial dimensions [62]. A Long Short-Term Memory (LSTM) network processes an input interpreted as an ordered set of values, where each value is addressed sequentially by an LSTM cell [63]. LSTMs belong to the family of recurrent neural networks (RNNs). In a ConvLSTM cell, all inputs, outputs, and states between steps are 3D tensors [64]. Compared to DECODE, SRST improved the JI and localisation error by 8% and 10 nm, respectively. However, SRST suffers from less accuracy at high fluorescence emitter densities.

Based on residual network and convolutional layers, CC-DeepLoc was recently proposed for tackling emitter localisation at high densities (~4 emitters/µm^2^) of emitters and a low SNR [65]. CC-DeepLoc slightly surpasses DECODE’s performance, effectively identifying most emitters. DECODE and SRST suffer from long training times when GPUs are not available. To tackle training and inference times, LiteLoc, a lightweight network consisting of a coarse feature extractor (CFE) and a fine feature extractor (FFE) (both are CNNs), implements an efficient CPU-GPU scheme for data loading and inference [66]. LiteLoc reduces by half the number of parameters needed by DECODE (from 2.20 M to 1.33 M) and increases computational efficiency by an approximate factor of two in terms of giga-floating points of operation (GFLOPs, which measure the number of billion floating-point operations needed to solve a specific task), thus reducing the computational cost from 170.46 to 71.08 GFLOPs. LiteLoc requires far fewer operations than DECODE. Still, it lacks the spatio-temporal feature extraction accomplished by SRST. LiteLoc can replace its CNNs with Bidirectional ConvLSTMs, although this may compromise the proposed CPU-GPU scheme. An alternative approach to achieving spatio-temporal feature extraction while maintaining computational efficiency would be to implement attention mechanisms [67], which could capture temporal dependencies without the computational overhead of ConvLSTMs.

### 2.2. Trajectory Linking via DL

Following molecule localisation [53,55], validated localisations must be linked to trajectories to study the dynamic behaviour of the probe-labelled molecules under investigation. Such a process is called trajectory linking (Figure 2d). Linear Assignment Problem (LAP) [50,51] and nonparametric Bayesian methods [52] are non-data-driven frameworks to link localisations to find trajectories. Although both methods are robust in high-density conditions, they present some weaknesses. LAP links trajectories optimising a cost function which needs to be specifically defined for each tracking application [50], whereas BNP-Track emphasises long periods of tracking (300 min) for 22 frames of 1500 pixels [52]. Trajectory linking with ML has been employed for cell tracking [53], but few ML-related applications are found in the case of molecular tracking. ML methods for molecular tracking can employ neural networks suitable for specific experimental conditions using synthetic datasets, bypassing user fine-tuning and slow inference. One of the most interesting conclusions from ref. [53] is that dynamics can be extracted from SMLM datasets using Graph Neural Networks (GNNs [68]) without requiring the proper trajectories. However, trajectory linking may still be needed to interrogate molecular processes like clustering at the single-track level in the temporal dimension. GNNs can be an attractive option to extract trajectories from SMLM datasets. The latter are transformed into graphs, whereby localisations are considered nodes, and these are linked via edges using spatio-temporal criteria [53,69]. Such criteria need to be carefully selected to avoid overwhelmingly large graphs when applied to SMLM datasets (which may contain more than 100,000 localisations, beyond what can be stored in the computer’s memory) [70].

Yet another approach is to employ Transformer-based networks [67]. Instead of processing each step sequentially as LSTMs do, the entire input sequence is generally processed in a single step. Thus, attention layers overcome the long- and short-term memory limitations of LSTMs, although they require more computational memory. An example of a Transformer-based network is the Motion Transformer Tracker (MoTT) [54]. First, a tree called a hypothesis tree (HT) is created, where the root node is a localisation from frame $t_{i}$, and consequent nodes are localisations that are temporally and spatially closer to the root node (Figure 2c). This tree holds multiple candidate tracklets (i.e., segments). Next, a Transformer using the already predicted track determines the probability of the next localisation being the following consecutive position in the same track; the algorithm iteratively continues predicting the succeeding steps of the trajectories (Figure 2d). Whereas LAP showed α = 0.557 (α measures the degree of matching between ground truth and estimated tracks) in high-density scenarios, the degree of matching of MoTT was α = 0.903 [54]. The disadvantage of this methodology is that it is limited to trajectory linking only. In contrast, SPTNet not only performs trajectory linking but also predicts the Hurst exponent (H) and diffusion coefficient (D) using raw images [55]. In other words, it provides detection, localisation, linking, and regression outputs in an end-to-end approach. In Section 5, we critically review ML methods for analysing the dynamics of trajectories.

Work referred to so far requires large, synthesised datasets to effectively train their neural networks. Recently, a Video Flow-Informed Neural Network (VFINN) [56] was proposed to accomplish high-density particle tracking without requiring ground-truth data. It relies on the concept that, given an ordered set of consecutive frames, the second frame is the distorted (“warped”) version of the first frame. The VFINN reduces the differences between the two frames, predicting an optical flow field (a matrix of the same size as the frames, where each pixel encodes the direction in which the first frame is shifted relative to the second frame). The VFINN processes the field to obtain particle localisations and trajectories. Although it is an elegant approach and bypasses synthetic data generation, it may not adequately capture very rapid diffusion phenomena, as molecules often move further than several pixels in a single frame, and the field may not register such shifts between pairs of frames. Considering additional temporal and spatial context beyond proximities would improve the performance of this network.

The use of FL methods to extract localisations from superresolution images is still unexplored. Although DL methods are generally more accurate, they lack the efficiency of FL methods in terms of training and inference speed. Ilastik is a graphical user interface (GUI) to label pixels in images and train FL models, relying solely on easy-to-extract features assigned to each pixel, such as colour and intensity [71,72]. Pixels from simulated images can be classified into those that include particle localisation and those that do not, from which prediction maps can be furnished and subsequently used to extract the final localisations.

## 3. Single-Molecule Trajectory Characterisation

**Figure 3:**
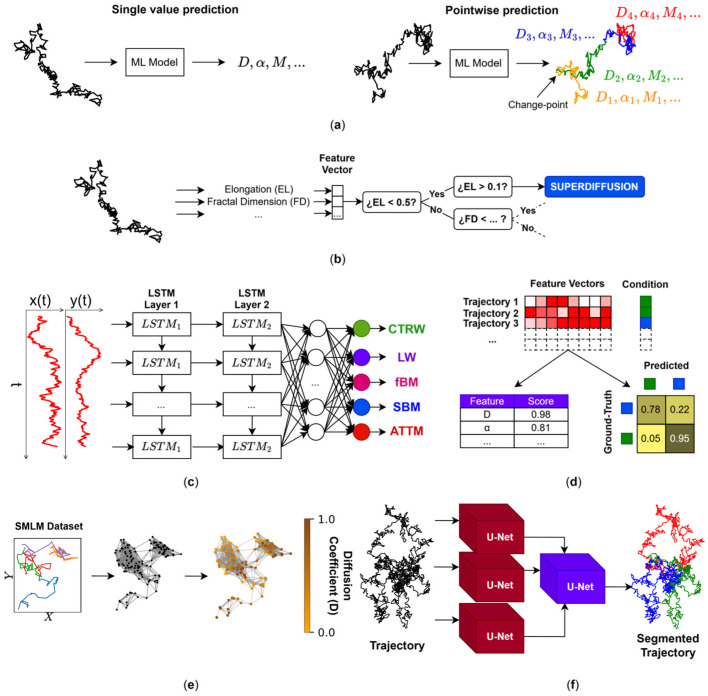
ML models in SMT analysis. (**a**) The scheme represents the difference between single-value and pointwise prediction of SMT behaviour. Whereas single-value prediction infers the value of parameters such as diffusion coefficient (D), anomalous exponent (α), or diffusion regime (“state”, M), pointwise prediction detects change-points (CPs) where such parameters change. The ellipse after M indicates that the models are not limited to only predict the D, α, and M. The colours used in pointwise prediction represent segments undergoing different diffusion regimes as predicted by the corresponding ML model. (**b**) Decision tree used to classify trajectories according to their features (elongation, fractal dimension, and long-time/short-time D ratio), which are stored in the feature vector. The decision tree classifies the trajectory according to basic decision rules. (**c**) Each trajectory position in the x and y axes is fed into an LSTM network. Each step is processed sequentially in the network. Finally, the output of the last LSTM layer is passed to an MLP layer to classify trajectories into the physical models of translational diffusion (categories) Continuous-Time Random Walk (CTRW), Levy Walk (LW), fractional Brownian motion (fBM), Scaled Brownian Motion (SBM), and Annealed Transient Time Motion (ATTM). (**d**) Fingerprint analysis consists of generating a feature vector for each trajectory and labelling it with the experimental condition to which it belongs. These vectors can then be used to train an ML model that quantifies how discriminable the trajectories are across experimental conditions via confusion matrices. In addition, the features within each vector can be ranked to identify which characteristics contribute most to the differences between the two conditions. (**e**) All trajectories within an SMLM dataset are converted into a graph following a specific criterion. Each node in the graph represents a localisation, and edges are created according to that criterion. A Graph Neural Network (GNN) then predicts the diffusion coefficient for each node. In this way, single-molecule dynamics can be analysed without requiring prior trajectory linking, since the graph-building criterion does not rely on trajectory reconstruction. (**f**) DeepSPT segments trajectories into several diffusion behaviours (indicated in red, blue and green) using a network composed of U-Nets. Table 2 compares different ML and DL methods suitable for trajectory analysis.

Methods built to characterise single-molecule trajectories are mainly divided into two categories: those that analyse trajectories (single-value) and those that consider every trajectory point pointwise (Figure 3a). The most widely employed tool to characterise single-molecule trajectories is mean-squared displacement (MSD) [73,74,75]. This classical approach has two main limitations. First, the accuracy with which MSD-based analysis extracts information from trajectories is strongly dependent on the trajectory length: as the trajectory length decreases, the uncertainty of the values extracted from the curve increases. Second, MSD curves are susceptible to noise, which can bias extracted parameter values such as the anomalous exponent (α). For pointwise analysis, window size strongly affects the precision with which dynamic parameters are determined [73]. Some works have proposed tackling the state classification of trajectories using Hidden Markov Models (HMMs) [76,77]. Both MSD and HMMs are surpassed by ML techniques that use simulated data for training [21,78,79]. Using simulated trajectories, ML methods can learn to classify trajectories into several categories (e.g., confined and non-confined) or infer real-valued dynamic parameters (e.g., α) without requiring sophisticated algorithms and statistical frameworks like those employed by traditional methods. Moreover, ML methods can explore non-linear relationships between different positions of a single trajectory and predict more accurately. In addition, ML methods can analyse beyond the current state of the trajectory (differing from HMM methods) as they capture long- and short-term correlations. Carlo Manzo and coworkers organised the First [21] and Second [80] Andi Challenge competitions in which the performance of several ML models were compared in their ability to predict single- or pointwise values of simulated trajectory dynamics, respectively. In this section, we will review the methods that participated in the two Andi Challenges, as well as those that were introduced during and after these competitions.

**Table 2:** Comparison of traditional and ML methods for trajectory analysis.

Method	Input	Strengths	Limitations
Mean Squared Displacement (MSD)	Trajectory positions	Simple and interpretable	Strongly length-dependent and sensitive to noise
		Extracts D and α	Biassed α estimation
Hidden Markov Models (HMM)	Trajectory positions or displacements	Probabilistic state classification	Model-dependent and limited flexibility
		Detects transitions	Surpassed by ML in performance
Decision trees	Engineered features	Interpretable inputs	Model-dependent
		Good generalisation	Difficult feature selection
		No GPU required	Raw coordinates unsuitable
FL-based neural networks	Engineered features	Stable across variable lengths	Difficult feature selection
		Computationally efficient	May underperform DL-based neural networks
Recurrent Neural Networks (RNN, LSTM, and Bi-LSTM)	Trajectory positions or displacements	Sequential nature of trajectories is considered	Slow training
		Handle uneven sampling	Length-dependent
		Strong regression performance	May underperform TCNs
Temporal Convolutional Networks (TCNs)	Trajectory positions or displacements	Faster training than LSTMs	Length-dependent
		Strong β/H prediction	Length-specific models are required
Trajectory-to-image representation (GAF)	GAF representations of trajectories	Strong classification accuracy	Computational expense
			Longer training
			Length-dependent
RNN + CNN Hybrid	Trajectory positions	Combined spatial feature extraction and temporal modelling	High computational cost
			Inefficient training due to LSTM component
Extreme Learning Machine	Engineered features	Very fast training	Shallow model
		Suitable for initial screening	Limited precision and generalisation
Autoencoders	Trajectory positions	Unsupervised method	Limited interpretability
		Anomaly detection	
		Latent representation learning	
Transformers	Trajectory positions	Capture long-range dependencies	Computationally demanding
		Strong generalisation	
Fingerprint analysis	Engineered features	Unsupervised discrimination of experimental conditions	Requires supervised methods
		Feature ranking	Depends on feature engineering
Graph Neural Networks (GNN)	Graph representation of localisations	Avoid trajectory linking	Graph construction critical
			Performance sensitive to transformation design
Sliding-window approaches	Overlapping sub-trajectories	Local prediction	High inference time
		No need for heterogeneous simulated trajectories	Window-size tradeoff between resolution and accuracy
			Unstable for short segments
Sequence-To-Sequence	Trajectory positions	Pointwise prediction	High model complexity
		No sub-trajectory splitting	Length-dependent
			Long training time
Change-point-based methods	Trajectory positions	Faster than sliding-window approaches	Class imbalance between positions with and without a change-point
		Explicit CP detection	Model-dependent
		Segment-wise inference	
Support Vector Machines (SVM)	Engineered features	Strong segmentation performance	Requires feature engineering
		Robust classification	Less scalable than DL
Bayesian Deep Learning	Trajectory positions	Provide uncertainty estimation	High computational cost

### 3.1. Andi Challenge: Single-Value Characterisation

The First Andi Challenge consisted of two main tasks: a prediction of β (task 1; T1) and the classification of trajectories into the diffusion models Continuous-Time Random Walk (CTRW) [23], fractional Brownian motion (fBM) [81], Levy Walk (LW) [82], Scaled Brownian Motion (SBM) [83], and Annealed Transient Time Motion (ATTM) [84] (task 2; T2).

#### 3.1.1. Predictions for Qualitative Analysis

Almost all the participants who beat T1 also beat T2. The new best competitor is a model that used HIVE-COTE and random forests, a special type of decision tree [85]. This decision tree was the first ML model applied to classify single-molecule trajectories using a Bayesian decision tree [86]. The algorithm first decides if trajectories are Brownian or confined motion according to the Bayesian Information Criterion (BIC). Those trajectories classified as confined pass through another decision tree to determine whether the confinement takes place in a harmonic or anharmonic potential [87] through the Akaike Information Criterion (AIC). The technique was applied to the characterisation of toxin receptor diffusion and its confinement portions at the cell membrane. Although such a decision tree is easy to interpret and to implement, it has three important drawbacks: (i) it is model-dependent (motion models must be analytically defined a priori), (ii) it does not take advantage of learning to automatically create the decision tree, and (iii) the number of models it considers (Brownian and confined) is very limited. A similar approach is followed by DiffusionLab, a model that allows decision-tree training but requires manual trajectory labelling, which requires prior knowledge of the most descriptive model for the trajectories [88].

Since having experimental ground-truth datasets is not feasible, simulations are leveraged. In ref. [89], a random forest model was created to classify trajectories with only three features (elongation, fractal dimension, and the long-time/short-time D ratio) into Brownian, subdiffusive, and directed diffusion (Figure 3b). A set of trajectories was simulated following the categories under consideration for each trajectory, and a set of features was extracted for each trajectory. Using these features, the decision trees classify trajectories. The approach attained accuracies of 86%, 97%, and 94% in correctly classified short (60 steps), middle (180 steps), and long (540 steps) trajectories, respectively. Interestingly, accuracy decreases with the increasing length of the trajectories, a trend diametrically opposed to that of most of the ML models for trajectory classification and regression, where accuracy increases with increasing trajectory length [21]. The decrease observed with the random forest model could be related to the selected features, which may hamper the discrimination of trajectories as their length increases. The same research group found that reducing and carefully choosing the number of features can help gradient boosting machines (GBMs) and random forests to improve their performance [90]. Evidently, feature selection is critical in FL. With XGB (a special case of a GBM [91]), trajectory classification can also be carried out with 22 features per trajectory as input [92]. For a deep analysis of feature selection in decision trees to tackle anomalous diffusion classification, the reader is referred to ref. [93]. Which are the most used parameters? Generally, features are based on the positions of the trajectories, like efficiency and straightness, whereas other parameters are extracted from the MSD, like α and D [93]. Most of these features are linear and exhibit ergodicity-breaking behaviour.

#### 3.1.2. Prediction for Quantitative Analysis

In the case of T1, the six best performances used DL models (where raw trajectories are used as input) except for two that used FL models (where features from raw trajectories are used for prediction [94,95]). This suggests that DL models are preferable for the prediction of quantitative parameters of single trajectories. The two top-performing FL methods were CONDOR [94] and Deep Learning followed by Moment Scaling Spectrum analysis (DL-MSS) [95]. CONDOR is a concatenation of Multi-Layer Perceptron (MLP) networks that extract 92 length-independent features from classical statistics per dimension and characterise trajectories by their β and corresponding theoretical model [94]. We have recently adapted this approach to classify confined sub-trajectories to analyse MINFLUX superresolution SMTs in live mammalian cells [96]. One of the main as yet unexploited advantages of CONDOR is that its inputs are interpretable and can be varied to check how the output varies to extract biological implications of the used features (e.g., measure the association of the predicted β values with the approximate entropy).

DL-MSS uses a bidirectional LSTM (Bi-LSTM [97,98]) to segment trajectories into different diffusion modalities; the resulting tracklets are then analysed by moment scaling spectrum (MSS; analysis of order moments higher than the second moment) to acquire dynamic parameters from each diffusion regime [95]. During the competition, this network was adapted to work with FL, adding a dense layer at the end of the network to predict β and use the displacement as input at each step of the trajectory, thus not exploiting the high order statistics offered by MSS. Adding more robust features to non-ergodic stochastic processes like multifractal spectral (MFS) features [99] can improve the accuracy of the prediction of dynamic properties [100]. However, even then FL does not perform better than DL-based architectures, especially LSTM networks [100].

DL models that best predicted β in T1 used RNNs [101,102], CNN [103], or RNN + CNN [104] setups. RNNs (like LSTMs) take advantage of the sequential nature of trajectories and offer a suitable approach for the analysis of SMT tracks. Bo and coworkers showed that RNNs can effectively characterise trajectories even when some points are missing or the experimental sampling has been uneven [105]. These authors subsequently presented an LSTM network [63] coined RANDI [101] that can decode the α coefficient and the theoretical model that best describes the trajectories of any dimension. Including a WaveNet encoder [106], Li and coworkers used such a layer for feature extraction; features are then analysed by LSTMs. Such an approach is critical as the WaveNet encoder consists of a stack of multiple (16) sequential layers, making the training procedure too slow. To accelerate training times, all the weights can be trained by a network called WadTCN, which includes a WaveNet encoder of eight layers, in trajectories of 25 steps, and reuse the weights of the whole network (except for the MLP layer) to subsequently train the network on longer trajectories [107]. Although length-specificity is not solved, this approach provides a framework for the feature extraction of trajectories that can be repurposed for segmentation and classification. Although practical, LSTMs were shown to perform more poorly than TCNs, which are based on learnable and causal (i.e., there is no leakage from the future to the past) convolutions [108].

Shechtman and coworkers [103] presented a multi-channel TCN to tackle the prediction of the Hurst exponent (H = β/2), which constitutes a more suitable parameter than β because H is a normalised (a good practice in ML) version of β (H only ranges between 0 and 1). Subsequently this network was employed to segment two-state trajectories into free and obstructed diffusion in an end-to-end approach [109]. With minimum pre-processing, trajectories could be segmented with a classification accuracy of over 90%. However, both approaches are length-dependent, i.e., a network must be generated for each length. A combination of convolutions and LSTMs was also explored [104]. However, LSTMs carry inefficient training due to their sequential nature.

#### 3.1.3. Aftermaths of the First Andi Challenge

The development of ML models to tackle single-value analysis of SMTs has steadily continued since the First Andi Challenge. Manzo and coworkers proposed a fast approach to deploy an Andi Extreme Learning Machine (Andi-ELM) via a shallow MLP to classify and predict the α exponent of trajectories, randomising weights in hidden layers, and training the output weights [110,111], considerably accelerating the training process. Andi-ELM is intended for initial analysis preceding more precise and time-consuming approaches. In addition, the number of parameters of MLPs is perhaps the highest among FL models, which may imply low predictive generalisation. As we will discuss further in this review, residual networks for anomalous diffusion decoding were proposed to tackle vanishing gradients [112], as implemented in ref. [107]. Additionally, CNNs created to analyse images can also be used to characterise trajectories by converting them into images using Gramian angular fields (GAFs): these matrices encode sequential data such that the inherent order of the sequence is not lost [113].

Supervised methods are not the only tool for characterising trajectories; unsupervised methods have also been developed for this purpose. In ref. [114], Manzo and coworkers developed a convolutional autoencoder (AE) [115] to detect whether diffusion is anomalous or not: the autoencoder learns to reconstruct trajectories from a low-dimensional encoding and, if the trajectory is reconstructed poorly (as judged by a metric like mean squared error), it is considered anomalous. Recently, the same research group developed a β-variation AE (β-VAE) [116] (also called disentangled variational autoencoder [117] to describe stochastic processes using just six artificial neurons for further simulation [118].

Hatzakis and coworkers introduced the use of fingerprints in SMT analysis [119,120]. A fingerprint is a vector containing single-value features (e.g., trajectory length). It is used to train neural networks to rank features according to their different outputs under varying experimental conditions. Although training NNs is necessary to accomplish the task, this is an unsupervised algorithm capable of detecting which characteristics differ in two (or more) trajectory datasets. Transformers can also be used to characterise trajectories in a “global” manner: ConvTransformer, an architecture that combines convolutions with Transformers, can tackle the regression and classification of anomalous diffusion [121]. Interestingly, these authors indicate that Transformers can help to discern which features are relevant and which are not. Hence, Transformers may break the inherent “black-box” model of DL architectures [121]. Regarding uncertainty predictions, Bayesian Deep Learning can be employed to estimate not only the model that best describes the trajectory along with its anomalous exponent but also the prediction uncertainty [122]. This approach can be extended to pointwise prediction [123].

#### 3.1.4. FL or DL?

FL demonstrated success in trajectory characterisation. However, its dependence on feature selection remains a bottleneck. Feature selection in FL is a laborious process in which a given subset of features is used for training and is compared with the accuracy of other subsets. In addition, the selection of cofounding variables may provoke overfitting and poor generalisation. Although practical, it is a memory- and time-consuming process based on trial and error [124]. The simplest way to bypass parameter selection is to directly feed trajectories into DL architecture, without feature extraction; in this case, the constituent layers are feature extractors. Can decision trees be employed to characterise trajectories using raw data input without extracting features (i.e., use decision trees as a DL method)? Indeed, RFs can be used to classify trajectories into different theoretical models and predict their α coefficient using normalised trajectories as input [125]. Although such RFs perform well on simulated and experimental trajectories, raw input is not suitable for decision trees. Because decision trees are based on binary decisions that partition the feature space, they are suited to structured, tabular data. In the case of raw trajectories as input, binary decisions are taken based on coordinates (which have no semantic meaning) instead of engineered features, making the implementation unsuitable.

CNNs have been employed to classify trajectories into physical models of anomalous, confined, directed and Brownian diffusion (Figure 3c) [126]. These authors compared their approach with GBM and RF, both FL methods, concluding that DL performs better (97.3%) than FL-based approaches (96.7%). However, FL approaches were 72 times faster (1 h) than DL (3 days) in their total processing time (data preparation, feature extraction, model tuning, training, and validation). DL had a better classification performance as trajectories reached Brownian behaviour and directed motion with low velocities. Lastly, decision trees outperform DL methods at generalising the classification of trajectories out of the training dataset (accuracy~20%): DL methods require a larger number of trainable parameters and perform very well on trajectories of the simulated data on which they were trained. However, they lack generalisability, performing less well on experimental datasets, since they are free to learn their own features during training instead of relying on a small set of user-defined features. The latter are also better at generalising than DL methods. Since features extracted using DL methods are length-dependent [107], FL-based networks outperform DL approaches in trajectory analysis when a wide range of lengths is present in the experimental trajectory. Another advantage of FL-based approaches is that they do not require GPU computing to attain acceptable levels of processing time. The prediction accuracy of both α and the theoretical model improved as the SNR increased (i.e., less noise) [21]. If noise is completely removed from trajectories, the F1 score increases to 0.84 in trajectories of 25 steps [107].

### 3.2. Andi Challenge: Dynamic Pointwise Prediction of Dynamics

Single-value dynamic characterisation focuses on predicting global properties of trajectories. However, since trajectories do not necessarily maintain constant dynamic behaviour throughout their lifetime, the predicted value often represents an average over the entire trajectory duration [127]. Thus, the pointwise prediction of dynamics requires the detection of temporal changes (change-point detection; CP) and prediction of physical properties at every trajectory point. The Second Andi Challenge assessed how well ML methods can detect changes in the α, D, and diffusional behaviour (e.g., confined or not) over time. Most of these ML models can be categorised into three main categories: sliding-window, sequence-to-sequence (Figure 3c), and CP-based inference (Figure 3d).

#### 3.2.1. Sliding-Window Approaches

Early methods for pointwise prediction relied on the use of average sliding windows, where trajectories are divided into overlapping sub-trajectories and each of these is fed into FL or DL models to perform a single-value inference. Subsequently, results for each single-point are averaged. Milhiet and coworkers developed a sliding-window and single-layer MLP approach to analyse confined, directed, or Brownian motion within trajectories of viruses and transmembrane proteins recorded using total internal reflection fluorescence (TIRF) microscopy [128]. The input data was the MSD of each consecutive 20-frame-long sub-trajectory [129]. The network predicted the probability of each point being in each state; the change between states was detected using a probability threshold. The same research group [89] added more features to their sliding-window decision tree to classify trajectories. Whereas tree-based qualitative segmentation of trajectories provided was 94.5% and 89.44% accurate for confined and directed motion, respectively, the accuracy of MLP methods was in each case 99% and 78%. The MLP-based analysis is sensitive to the length of the segments; short state segments (around 30 frames) decrease the accuracy for confined and directed motion to 74% and 72%, respectively. Decision trees were more stable in this respect. One advantage of sliding-window approaches is that they do not require simulation of heterogeneous trajectories because prediction is local rather than global. But they suffer from long inference times because, as trajectories of l steps are divided into overlapping segments of length w, l−(w−1) predictions are made. It would be convenient to infer pointwise properties in a single step. To attain higher resolution, window lengths should be as short as possible; however, this reduces prediction performance, since shorter segments have lower accuracy and lead to worse predictions [21]. Support Vector Machines (SVMs [130]) have also been employed to segment trajectories into different diffusion behaviours with a careful feature selection, achieving better performance than average sliding-window methods [131]. Using DL, an MLP was proposed to predict the Hurst exponent (H = α/2) of fractional Brownian motion (fBM) for every trajectory point [78]. However, the number of predictions required was still high.

#### 3.2.2. Sequence-to-Sequence Approaches

An approach to improve the analysis of overlapping segments is to treat the pointwise prediction as a sequence (trajectory)-to-sequence (output of the same length as the trajectory) prediction. Trajectories segmented in a sequence-to-sequence fashion into different D, α, and state values in a single iteration do not rely on the use of sub-trajectories. One way to accomplish the segmentation task is to extend the network of ref [78]. However, since these authors employ an MLP, the number of weights would exponentially increase with the addition of the same number of steps as the output neurons. Moreover, MLPs are barely able to analyse sequential data [132].

A widely used architecture for segmentation in image processing is U-Net, which uses convolutional layers to return an image of the same size with predictions in each pixel [58]. Because it is mainly composed of 2D CNNs, it can be employed with TCNs, preferably for trajectory data applications. U-Net was recently used by Huang and coworkers [133] in combination with WadNet [102,106]. However, the repetition of a WadNet layer within the proposed U-Net extends training time and deters network efficiency. Moreover, change-point detection is proposed only in the α dimension. A stack of U-Nets in combination with DeepSPT was also proposed to qualitatively characterise trajectories [120]. DeepSPT resolves the problem of costly training times using only convolutional operations like fingerprints [119]. Using LSTMs, Bo and coworkers developed an LSTM to point-wise predict D and α and infer CPs using another network [134]. A similar approach was followed by Haidari and Kaipandis using BI LSTM (although CP was performed via traditional methods) [135].

Transformers [67] have also shown segmentation capabilities. A Transformer-based network (STEP) was developed to pointwise predict α and D: the two parameters were assigned to all points of the trajectory [136]. Hence, STEP can reveal how dynamic properties change through time in a data-driven approach. One interesting property of STEP beyond training data is that it generalises well in other theoretical models: training with Brownian trajectories with varying D is enough to reveal D in other theoretical models (e.g., Scaled Brownian Motion (SBM)). However, change-point detection of trajectories is carried out with linearly penalised segmentation (PELT) [137], which detects segments based on their mean value (see review on offline change-point detection in [138]) limiting change-point detection when one variable is constant and the other one changes. Manzo and coworkers developed MAGIK (Motion Analysis through GNN Inductive Knowledge): a GNN based on geometric DL, enhanced by attention-based mechanisms to analyse dynamic properties extracted from time-lapse microscopy datasets [53]. MAGIK performance was benchmarked on the trajectory linking task, though the authors showed that it is neither necessary to extract trajectories from the datasets nor to perform the actual linking to analyse cellular or molecular dynamics. Object features are handled by MAGIK with geometric priors, and the network can be applied to SMLM datasets, as exemplified with lipids and receptors in the plasma membrane of live cells. However, the transformation into SMLM datasets needs to be carefully designed as it limits prediction performance [70].

The above works rely on defined physical models in which trajectories are simulated for network training. Through unsupervised learning, Deep-SEES reconstructs trajectories using Variation AutoEncoders [139]. Instead of predicting a latent vector (belonging to $R^{20}$), these networks calculate the parameters of a specific distribution where the latent vector is sampled. They gather the latent vectors of all sub-trajectories and then observe the distribution of the vectors in a reduced space using t-SNE. A clustering detection algorithm is applied next to the latent vector. Each identified cluster is a distinctive diffusive behaviour.

#### 3.2.3. Change-Point (CP)-Based Pointwise Prediction

Detecting CPs is not the same as inferring dynamic properties using segments of trajectories obtained with detected CPs. CP detection also differs from sliding-window analysis because the number of sub-trajectories to analyse is lower. CP-based analysis is considerably faster as it only requires a few sub-trajectories for prediction. In the sequence-to-sequence approach, CPs are detected after the prediction of physical parameters. CP-based pointwise predictions detect the CPs in which diffusion changes and then predict each segment’s single values. The first work to tackle CP prediction in an end-to-end approach involved WadTCN [107]. The combination of TCNs and WadNet in a supervised method was used to successfully classify trajectory points into CPs or non-CPs. It is worth noting that supervised CP detection is considered a class imbalance problem [140], to alleviate which the loss selected during training is crucial. Focal loss or weighted binary cross-entropy may be employed to deal with this problem [141]. If probabilities are predicted, a decision threshold should be determined with algorithms like GHOST [142]. Using DL and FL, CINNAMON [143] detects CPs and predicts H and D for each segment. However, temporal linear changes in D may pass undetected (as does SBM). Attention-based networks can detect such linear changes in a single step [136]. With similar objectives, an LSTM network was proposed to segment trajectories into two alternative states: Brownian and anomalous diffusion [144]. The limitation of the latter work is that only one CP can be predicted, limiting multiple segmentation within a single trajectory.

## 4. Diffusion Mapping

Pix2D is an ML-enabled software that works on stacks of single-molecule images as input and, by exploiting the otherwise undesired motion blur resulting from the convolution of motion with the PSF, generates a CNN model that calculates a diffusion map as its output [145]. Pix2D excelled with traditional methods like maximum likelihood estimation (MLE) algorithms [146]; however, an obligatory requisite is that single molecules do not overlap, thus hindering analysis of dense samples. Aiming at the same as Pix2D, Manzo and coworkers developed a supervised geometric DL method to decode diffusion on the spatial dimension that employs a graph neural network enhanced by attention-based constituents, taking the entire SMLM dataset as input [53]. This series of methods does not provide a continuous diffusion map. Instead, the maps are discretised via “bins”, providing a D for single points in space.

As stated in the Introduction, to obtain a desired function f, most ML methods rely on optimising a cost function through manipulation of a defined set of parameters. Other, less commonly used methods assume that the function f is smooth and belongs to a broad set of possible functions. Modelling a distribution over functions, these approaches consider that f is the most probable function resulting from the training data [147]. What if the data is an SMLM dataset and the function is the D as a function of 2D coordinates? This is precisely where DiffMAP-GP, an unsupervised Bayesian method, comes in [148]: it takes the SMLM datasets and tests spatial points to build f. DiffMAP-GP has been applied to learn about the diffusion coefficient of membrane proteins. Although practical, it is time-consuming when the number of localizations in a region of interest exceeds ~50,000, as is often the case with results from superresolution techniques like MINFLUX [20,96].

## 5. Conclusions and Prospects

DL-based methods are convenient tools for localising and validating single-molecule data in superresolution experiments and for linking such validated localisations to the study of biological molecule trajectories. A drawback of DL methods is their slow training and their very high hardware demand (e.g., GPUs are needed to achieve acceptable speed). In contrast, FL-based techniques on low-cost hardware setups are considerably faster to train than DL methods but have so far not been implemented for particle localisation and trajectory linking. Whereas DL automatically extracts features from images to analyse SMTs, FL needs pre-defined feature extractors, which can be hard to define in many experimental situations when the input sample is an image. Lastly, the detection of sub-ROIs as in ref. [49] can accelerate the detection of localisations as the area under analysis is much smaller than the entire image. Sub-ROI analysis can be improved via object detection networks such as DeepBlink [149] using “you only look once” (YOLO) networks [150]. However, manual annotation is still needed.

Most of the methods reviewed in this work involve superresolution techniques with limited temporal resolution imposed by the camera-based acquisition (~10 ms). Currently, this temporal barrier has been surpassed by avalanche diode photon-counting nanoscopic methods like MINFLUX (<1 ms) [20,25,96,151], but these new developments are only beginning to impact the field of ML. Recently, an unsupervised algorithm to distinguish MINFLUX mobile from immobile trajectories was developed [152]. This work differs from analytical approaches (e.g., discrimination based on the radius of gyration R_g_ [153]) and needs to be extended to other diffusion models in the current experimental realm, in which molecules do not necessarily exhibit Brownian (random) motion.

FL-based approaches seldom tackle the relationship between inputs and outputs in the characterisation of single-molecule trajectories. Interrogating input/output relationships has two main advantages: (i) new relationships are found between physical variables, and this can help to gain insight into the biophysical implications of the findings, and (ii) the underlying structure of prediction models is made apparent, thus disclosing their rationale. However, such interrogation should be carefully addressed with a parameter selection approach to prevent including confounding variables that lead to spurious correlations. As indicated in Section 3.1, another aspect in ML-based SMT analysis that has not been addressed is noise. Efforts should be directed toward reducing noise in the samples as a pre-processing step. However, this is not a trivial task, as trajectories may become smoothed during denoising, altering the original dynamics.

In image processing, cGANs are widely employed to increase the spatial resolution of superresolution datasets [154,155,156,157,158]. Although cGANs have been widely employed for image-related tasks, they can also be applied to the analysis of time-series data. The main advantage of cGANs is that they do not rely on task-related loss functions (like MSE). Instead, the adversarial training process (based on the concept of Nash’s local equilibrium) enables the neural network to learn effectively. This approach can be leveraged to localise molecules and to quantify single-valued and pointwise dynamic predictions in trajectory analyses [159,160].

It is important to note that most of the discussed methods employ simulated, synthetic data to train models. Although practical, this may affect their broader applicability if simulations are not well-suited to specific applications. Some researchers may select simulation parameters (e.g., time between consecutive trajectory points) that are not representative of the experiment to be analysed, rendering the trained models unsuitable for these purposes. Simulations can be conveniently adapted ad hoc, however, to re-train the entire model or fine-tune pre-trained models (if available). Another aspect of relevance is model validation. ML methods are first validated using simulated data to assess their performance under controlled conditions. Subsequently, they are applied to experimental datasets, the results are statistically compared with those reported in previous studies, and differences quantitatively estimated. An alternative is to reproduce specific behaviours, such as free diffusion, in real experiments to establish reference parameters for validation; however, more complex diffusion modes, such as confinement, are considerably more difficult to replicate and pose additional challenges for validation using solely experimental data.

Finally, and from a more general perspective, foundational models like LLMs are increasingly being applied in industry and in academia outside the biomolecular field, especially in the creation of agents (which may include models other than LLMs) to solve complex tasks. Access to LLM models like GPT-5 and Claude Sonnet 4.5 is opening the possibility of obtaining results through prompts, i.e., prompt engineering, defined as engineered texts describing a task to accomplish in a human-readable approach [161]. With specific prompts, LLMs can for instance analyse time series data [162,163], though the analysis of SMT data using LLMs is currently unexplored, presenting an opportunity to undertake the analysis of biology-related time series through natural language, despite LLMs being slower than non-language-related models.

## Figures and Tables

**Table 1:** Comparison between traditional and ML methods for single-molecule localisation and trajectory linking.

Method	Input	Strengths	Limitations
PSF Gaussian fitting	Sub-regions of interest (ROI)	Accurate when assumptions hold	Sensitive to model assumptions
		Suitable for sparse emitters	Prone to user bias
			Not suitable at high molecule density
			Does not perform trajectory linking
CNN-based localization [49]	Sub-regions of interest (ROIs)	Resistant to noise (RMSE ≈ 1 pixel at SNR = 1)	Does not account for blinking across frames
		Independent of emitter density	Limited multi-molecule handling
			Does not perform trajectory linking
Linear Assignment Problem (LAP) [50,51]	Localization coordinates	Robust to high-density conditions	Does not reach theoretical optimum
		Considers movement heterogeneity, gap closing, and merging and splitting of tracks	Cost functions must be specifically adjusted for the tracking purpose
Bayesian nonparametric track (BNP-Track) [52]	Consecutive frames	Computational cost scales linearly with number of frames, pixels, and total emitters	Slow inference in standard desktop computers
		Free from manual tuning	
GNN-based trajectory extraction [53]	Graph representation of SMLM localizations	Can extract dynamics without explicit trajectories	Graph size can become computationally impossible
		Flexible graph modelling	Requires careful graph building criteria
Transformer-based network (MOTT) [54]	Hypothesis tree from localisations	Models long/short-term dependencies via attention	Limited to linking
		Iterative prediction	High memory usage
End-To-End DL Network (SPTNet) [55]	Consecutive frames	Detects localization, does trajectory linking and predicts dynamical parameters (e.g., diffusion coefficient)	Requires large synthetic datasets
Optical flow-based DL (VFINN) [56]	Consecutive frames	Does not require ground-truth synthetic data	May fail for rapid diffusion (large inter-frame displacements)
		Elegant optical flow formulation	Limited temporal context

## Data Availability

No new data were created or analyzed in this study.

## Abbreviations

The following abbreviations are used in this manuscript:

ML	Machine Learning
FL	Feature-based learning
AI	Artificial Intelligence
HT	Hypothesis Tree
DL	Deep Learning
CNN	Convolutional Neural Network
SMLM	Single-Molecule Localization Microscopy
STED	Stimulated Emission Depletion
PALM	Photo-Activated Localization Microscopy
STORM	Stochastic Optical Reconstruction Microscopy
PSF	Point-Spead Function
ROI	Region of Interest
RMSE	Root Mean Square
SNR	Signal-to-Noise Ratio
LSTM	Long Short-Term Memory
RNN	Recurrent Neural Network
GNN	Graph Neural Network
SMT	Single-Molecule Tracking
SPT	Single-Particle Tracking
MSD	Mean Squared Displacement
HMM	Hidden Markov Model
TCN	Temporal Convolutional Network
RF	Random Forest
GBM	Gradient Boosting Machine
XGB	Extreme Gradient Boosting
Bi-LSTM	Bidirectional LSTM
AE	Autoencoder
VAE	Variational Autoencoder
CP	Change-Point
TIRF	Total Internal Reflection Fluorescence
SVM	Support Vector Machine
cGAN	Conditional Generative Adversarial Network
MLE	Maximum Likelihood Estimation
MINFLUX	MINimal Fluorescence emission FLUXes
YOLO	You Only Look Once
GPU	Graphics Processing Unit
CPU	Central Processing Unit
GFLOPs	Giga Floating Point Operations
MLP	Multi-Layer Perceptron
